# Supplementary material for: Strategies for synthesis of Prussian blue analogues
Source: R Soc Open Sci. 2021 Jan 13;8(1):201779. doi: 10.1098/rsos.201779 (PMC7890497; doi:10.1098/rsos.201779)
Supplement: Supporting experimental data [file rsos201779supp1.pdf]

Supporting information

Strategies for Synthesis of Prussian Blue Analogues

Solveig Kjeldgaard[ab], Iulian Dugulan[c], Aref Mamakhel[b], Marnix Wagemaker[c], Bo Brummerstedt Iversen[b] and Anders Bentien[a]

[a] Department of Engineering, Aarhus University
[b] Department of Chemistry, Aarhus University
[c] Department of Radiation Science & Technology, Technical University Delft

Powder X-ray diffraction

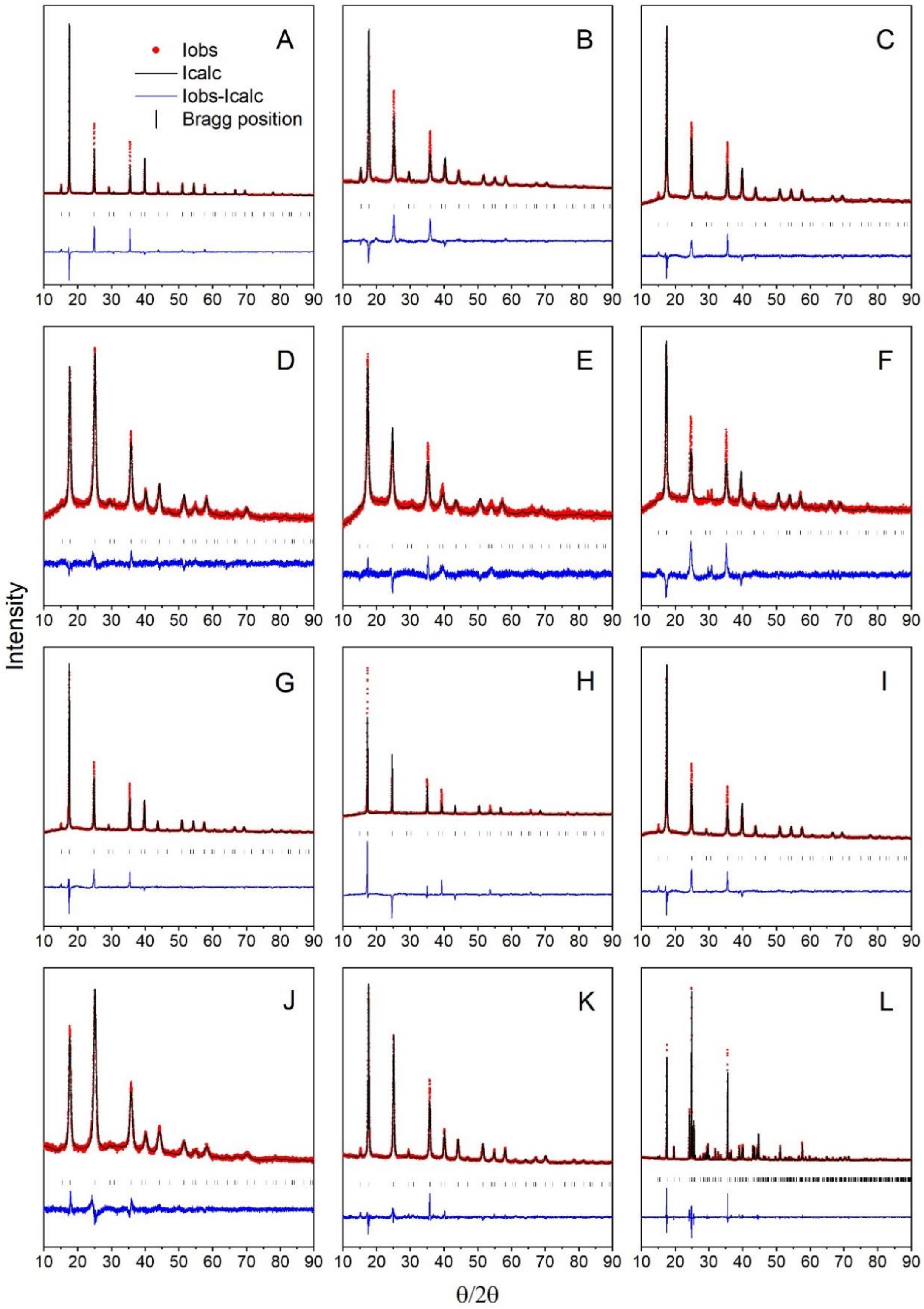

Figure S1: Rietveld refinement

Table S1: Crystallographic details

A	Space group	*Fm-3m*
	Number of data points	7995
	Number of background points	27
	Number of fitted parameters	38
	RBragg	18.5
	Rf	9.16
B	Space group	*Fm-3m*
	Number of data points	7995
	Number of background points	24
	Number of fitted parameters	34
	RBragg	17.9
	Rf	9.91
C	Space group	*Fm-3m*
	Number of data points	11243
	Number of background points	16
	Number of fitted parameters	27
	RBragg	11.7
	Rf	6.79
D	Space group	*Fm-3m*
	Number of data points	11243
	Number of background points	15
	Number of fitted parameters	27
	RBragg	4.02
	Rf	4.85
E	Space group	*Fm-3m*
	Number of data points	11243
	Number of background points	14
	Number of fitted parameters	21
	RBragg	8.65
	Rf	6.77
F	Space group	*Fm-3m*
	Number of data points	11243
	Number of background points	15
	Number of fitted parameters	22
	RBragg	16.6
	Rf	8.92
G	Space group	*Fm-3m*
	Number of data points	11243
	Number of background points	17
	Number of fitted parameters	28
	RBragg	9.99
	Rf	7.90

H	Space group	*Fm-3m*
	Number of data points	11243
	Number of background points	15
	Number of fitted parameters	22
	RBragg	40.5
	Rf	21.7
I	Space group	*Fm-3m*
	Number of data points	11243
	Number of background points	22
	Number of fitted parameters	33
	RBragg	14.7
	Rf	9.15
J	Space group	*Fm-3m*
	Number of data points	7995
	Number of background points	12
	Number of fitted parameters	24
	RBragg	3.04
	Rf	2.76
K	Space group	*Fm-3m*
	Number of data points	7995
	Number of background points	20
	Number of fitted parameters	31
	RBragg	5.53
	Rf	4.80
L	Space group	$P2_1/c$
	Number of data points	7995
	Number of background points	24
	Number of fitted parameters	43
	RBragg	6.28
	Rf	6.07

Transmission electron microscopy

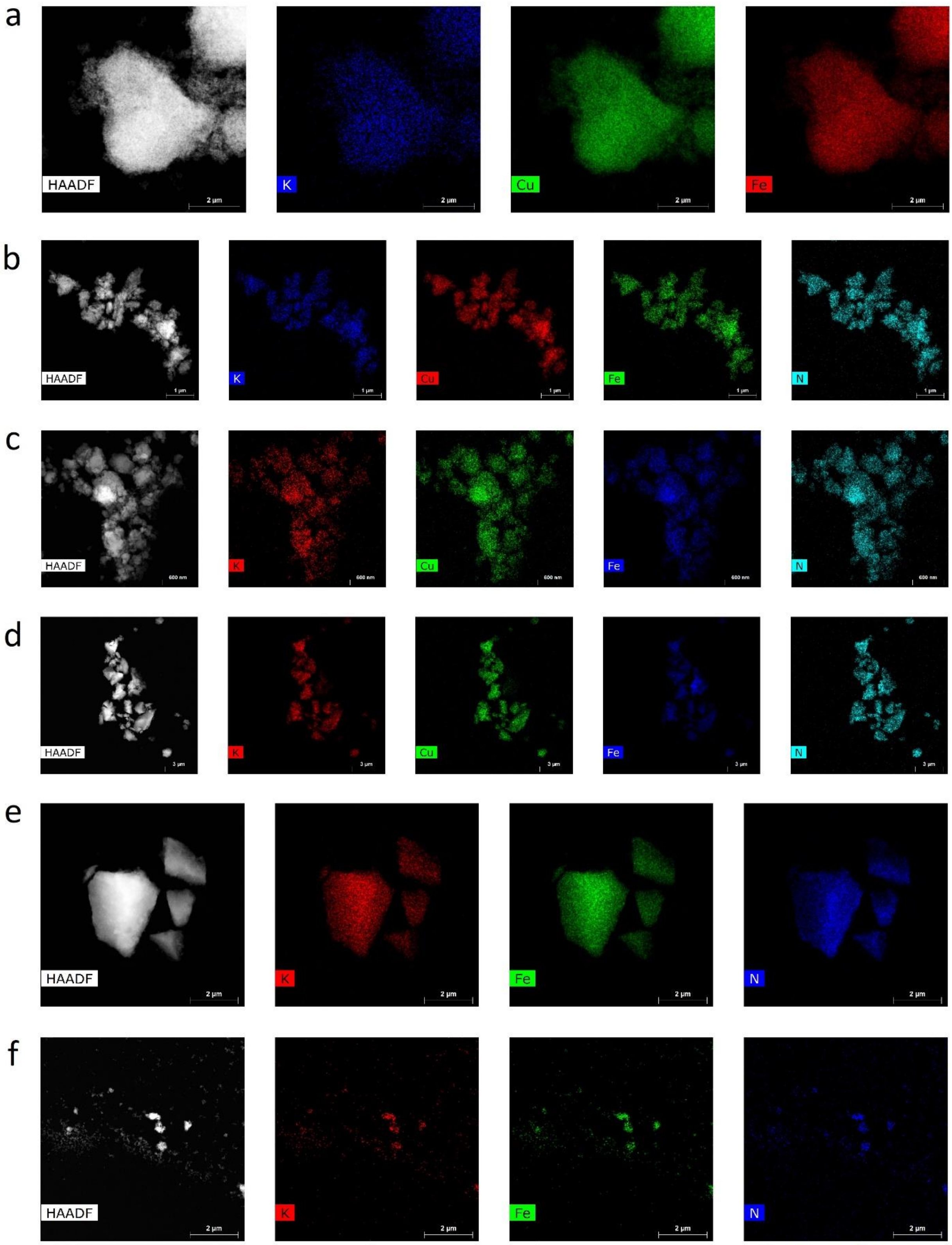

Figure S2: STEM-EDS

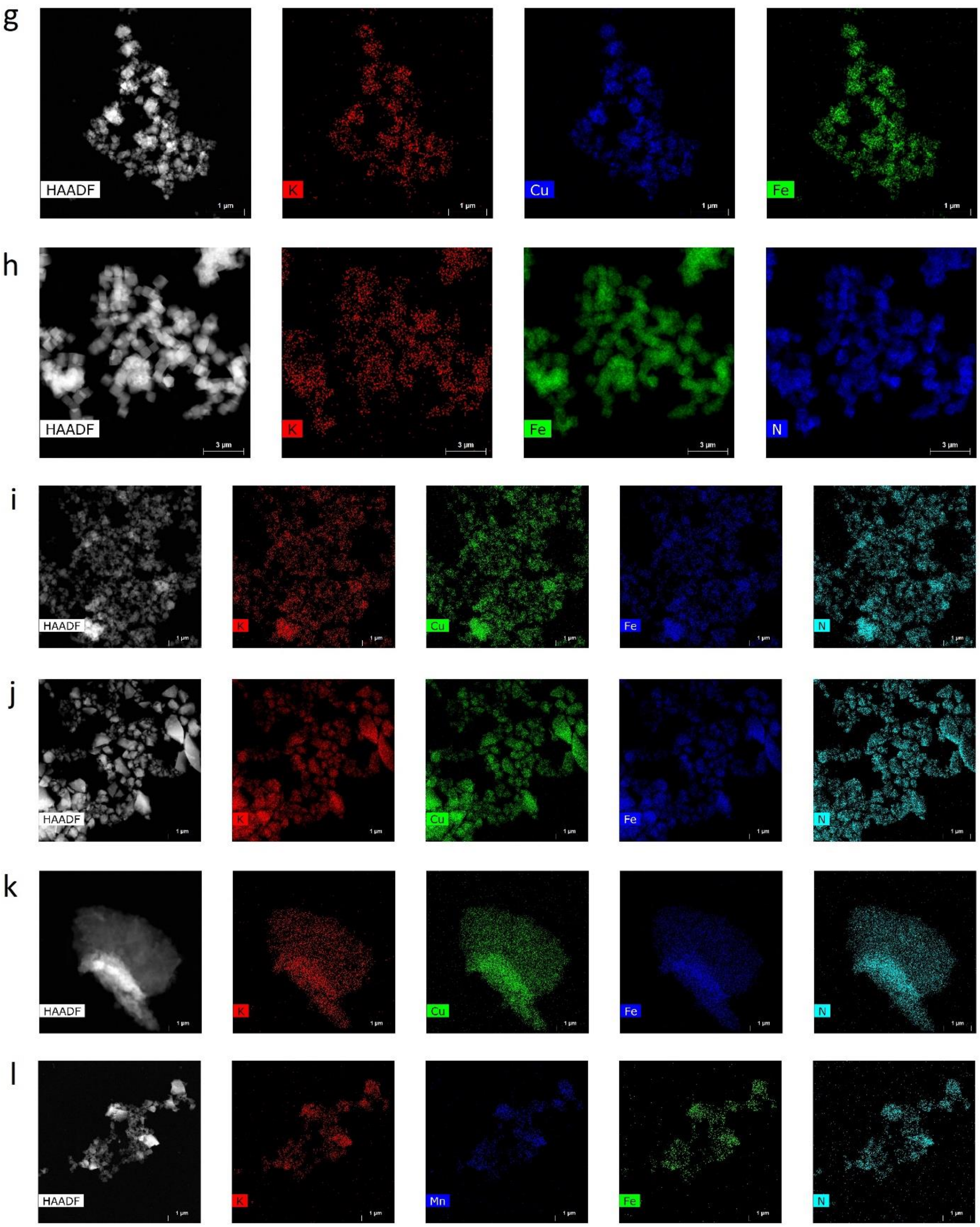

Figure S3: STEM-EDS

Energy dispersive spectroscopy (STEM-EDS)

It has been suggested that PBAs grown in the presence of excess chloride may contain Cl in the framework.[1] EDS spectra of sample G, Cu[Fe] precipitated in the presence of excess KCl, shows no Cl signal, indicating that Cl is not included in the framework.

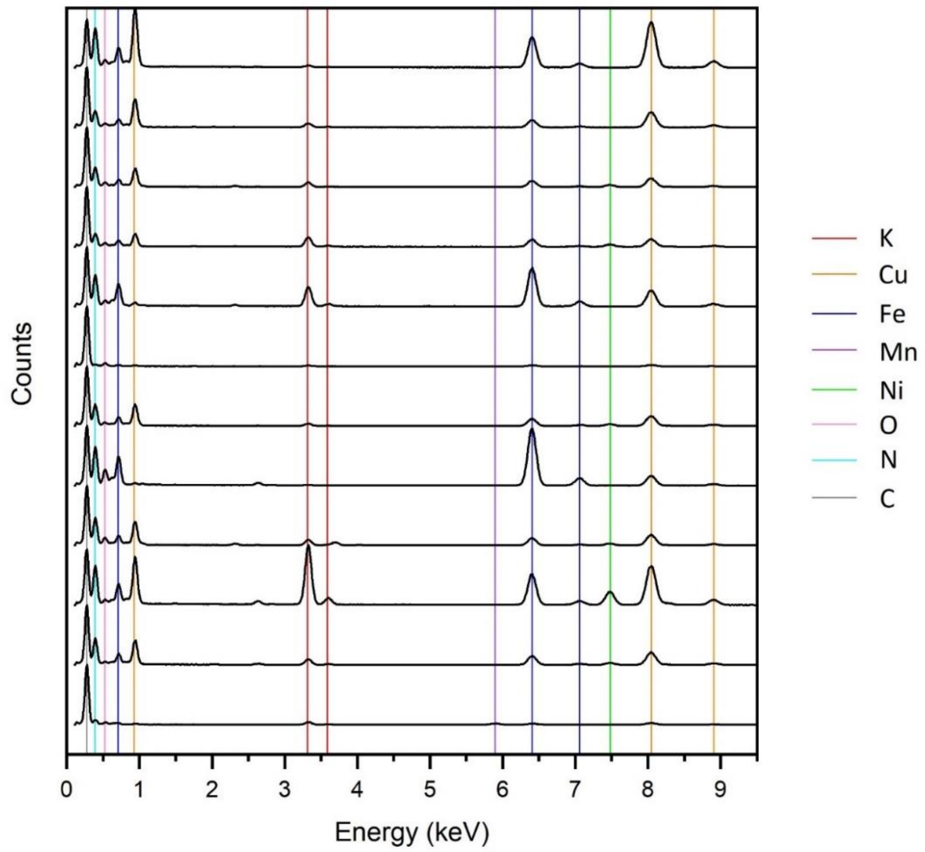

Figure S4: EDS spectra

Table S2: EDS table

Element #	Element	Line	Energy (keV)
6	C	Kα1,2	0.277
8	O	Kα1,2	0.5249
14	N	Kα1,2	0.3924
19	K	Kα2	3.3111
19	K	Kα1	3.3138
19	K	Kβ1,3	3.5896
25	Mn	Kα1	5.8988
28	Ni	Kα2	7.4609
28	Ni	Kα1	7.4782
29	Cu	Lα1,2	0.9297
29	Cu	Kα2	8.0278
29	Cu	Kα1	8.0478
29	Cu	Kβ1,3	8.9053

Mössbauer spectroscopy

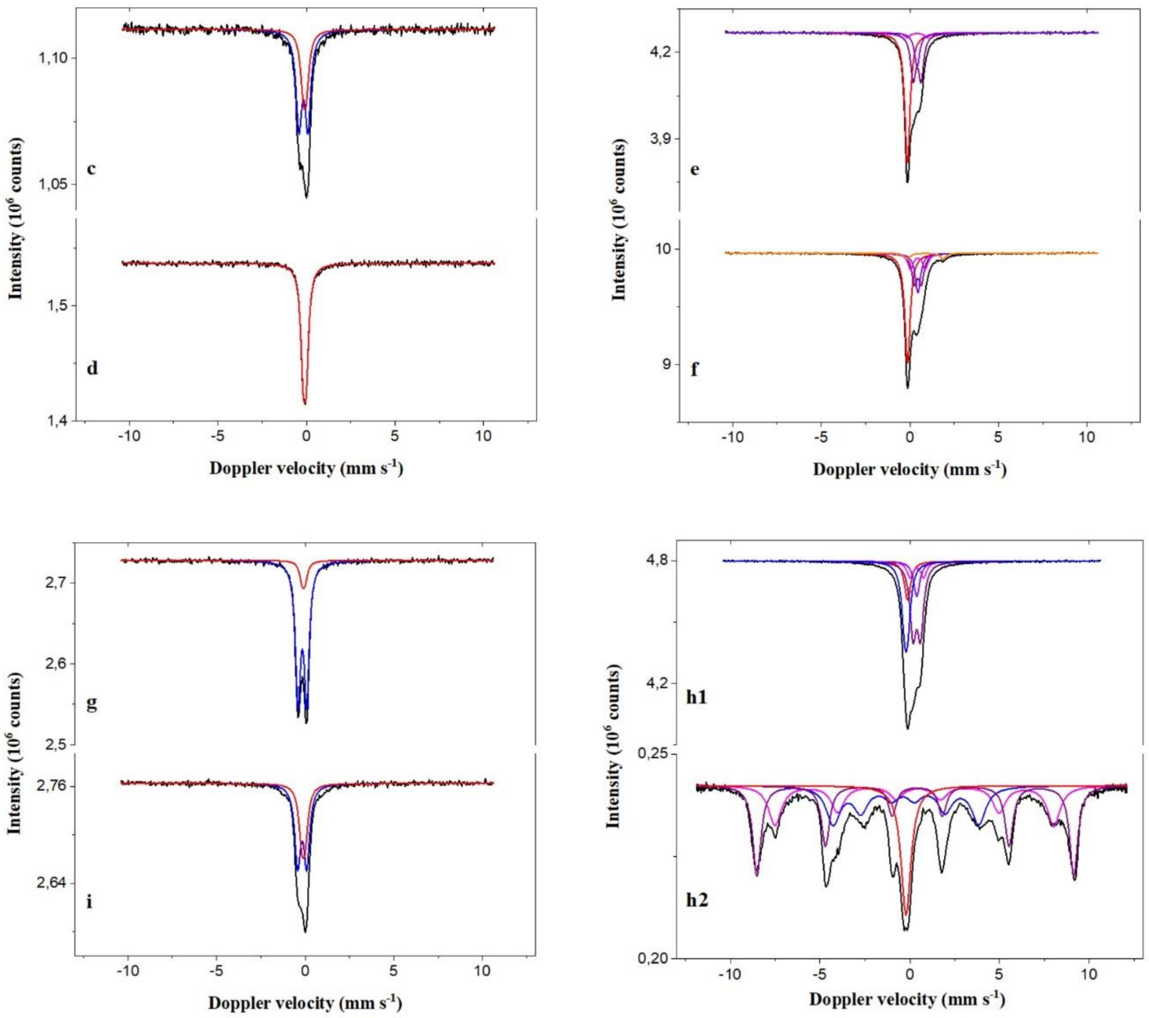

Figure S5: Mössbauer spectra obtained at 300K except h2 at 4.2K.

Mössbauer spectra of samples C-I were measured at 300 K. The fits have been constrained to a single linewidth (Γ) for all doublets, as Grandjean *et al.*[2] Grandjean *et al.* suggest that the use of different linewidths for the single iron(II) doublet and single iron(III) doublet might conceal variations in the local iron(III) electronic environment

Prussian blues are mixed valence compounds. The single quadrupole doublet with an isomer shift varying from -0.09 to -0.14 mm/s results from LS Fe(II) ions coordinated to six carbon. The three doublets with an isomer shift of approximately 0.40 mm/s result from HS Fe(III) ions coordinated to six nitrogen.

Mössbauer spectral parameters vary somewhat with temperature. The temperature dependence of the fitted parameters were investigated by Grandjean *et al.*,[2] indicating that the temperature dependence agrees well with the Debye model.

H1 and H2 is the same compound, measured at 300 K (H1) and 4.3 K (H2). The quadrupole doublets become magnetic sextets at low temperature.

Table S3: Mössbauer fitted parameters

Sample	T (K)	IS (mm/s)	QS (mm/s)	Hyperfine field (T)	Γ (mm/s)	Phase	Spectral contribution (%)
C	300	-0.09	0.17	-	0.39	LS Fe^{II}	34
		-0.17	0.50	-	0.39	LS Fe^{III}	66
D	300	-0.09	0.18	-	0.33	LS Fe^{II}	100
E	300	-0.14	0.00	-	0.35	LS Fe^{II}	50
		0.41	0.38*	-	0.35	HS Fe^{III}	50
F	300	-0.13	0.00	-	0.36	LS Fe^{II}	44
		0.45	0.38*	-	0.36	HS Fe^{III}	51
		0.87	2.00	-	0.36	HS Fe^{II}	5
G	300	-0.09	0.17	-	0.34	LS Fe^{II}	11
		-0.16	0.49	-	0.34	LS Fe^{III}	89
H1	300	-0.12	0.00	-	0.41	LS Fe^{II}	11
		-0.20	0.16	-	0.41	LS Fe^{III}	30
		0.40	0.39*	-	0.41	HS Fe^{III}	59
H2	4.2	-0.20	0.00	-	0.67	LS Fe^{II}	15
		-0.29	0.17	25.2	0.94	LS Fe^{III}	25
		0.37	-0.22	48.3	0.86	HS Fe^{III}	25
		0.37	-0.11	54.9	0.55	HS Fe^{III}	35
I	300	-0.09	0.19	-	0.39	LS Fe^{II}	37
		-0.17	0.51	-	0.34	LS Fe^{III}	63

Experimental uncertainties: Isomer shift (IS): ± 0.02 mm/s; quadrupole splitting (QS): ± 0.02 mm/s; line width (Γ): ± 0.03 mm/s; spectral contribution: ± 3 %.
*Average quadrupole splitting value (as in Grandjean *et al.*[2])

Stoichiometry of Fe[Fe] samples

The stoichiometry of Fe[Fe] samples is determined using Mössbauer spectroscopy data. The LS/HS ratio provides the number of vacancies, and potassium content is determined by charge balance.

Example calculation, sample H: $K_xFe^{III}[Fe^{III/II}(CN)_6]_y$

From Mössbauer: 40% Fe is low spin: C-Fe, 60% Fe is high spin: N-Fe

Y : 40/60 *100 = 67 %

X : 3 + (0.67*15/40)*2 + (0.67*25/40)*3 – 0.67*6 = 0.74

The stoichiometry is therefore $K_{0.74}Fe^{III}[Fe^{III/II}(CN)_6]_{0.67}$

References

1 D. E. Stilwell, K. H. Park and M. H. Miles, *J. Appl. Electrochem.*, 1992, **22**, 325–331.

2 F. Grandjean, L. Samain and G. J. Long, *Dalt. Trans.*, 2016, **45**, 18018–18044.
